# An interpretable DIC risk prediction model based on convolutional neural networks with time series data

**DOI:** 10.1186/s12859-022-05004-2

**Published:** 2022-11-08

**Authors:** Hao Yang, Jiaxi Li, Siru Liu, Mengjiao Zhang, Jialin Liu

**Affiliations:** 1 Information Center, West China Hospital, Sichuan University, Chengdu, China; 2 Department of Clinical Laboratory Medicine, Jinniu Maternity and Child Health Hospital of Chengdu, Chengdu, China; 3 Department of Biomedical Informatics, Vanderbilt University Medical Center, Nashville, TN USA; 4 Department of Medical Informatics, West China Medical School, Sichuan University, No. 37 Guoxue Road, Chengdu, 610041 China

**Keywords:** Disseminated intravascular coagulation, Prediction, Machine learning

## Abstract

Disseminated intravascular coagulation (DIC) is a complex, life-threatening syndrome associated with the end-stage of different coagulation disorders. Early prediction of the risk of DIC development is an urgent clinical need to reduce adverse outcomes. However, effective approaches and models to identify early DIC are still lacking. In this study, a novel interpretable deep learning based time series is used to predict the risk of DIC. The study cohort included ICU patients from a 4300-bed academic hospital between January 1, 2019, and January 1, 2022. Experimental results show that our model achieves excellent performance (AUC: 0.986, Accuracy: 95.7%, and F1:0.935). Gradient-weighted Class Activation Mapping (Grad-CAM) was used to explain how predictive models identified patients with DIC. The decision basis of the model was displayed in the form of a heat map. The model can be used to identify high-risk patients with DIC early, which will help in the early intervention of DIC patients and improve the treatment effect.

**Supplementary Information:**

The online version contains supplementary material available at 10.1186/s12859-022-05004-2.

## Introduction

“Disseminated intravascular coagulation (DIC) is an acquired syndrome characterized by the intravascular activation of coagulation with loss of localization and arising from different causes. It can originate from and cause damage to the microvasculature, which if sufficiently severe, can produce organ dysfunction” (defined by the International Society on Thrombosis and Haemostasis, ISTH) [1]. DIC is often regarded as a serious, life-threatening and complex clinical condition [2, 3], which is elicited by malignancies, serious infections, trauma, obstetric diseases, liver diseases, etc. [4]. Mortality is remarkable to increase in patients who develop DIC, and the risk of death is doubled in critically ill patients [5].

The diagnostic criteria, the DIC score (by ISTH scoring system) is widely used in clinical practice [6], similar scoring algorithms have been developed and widely evaluated in various countries, like the Japanese Ministry of Health and Welfare (JMHW) and Japanese Association for Acute Medicine (JAAM) [7]. However, no single clinical or laboratory test has adequate sensitivity and specificity to confirm or reject a diagnosis of DIC [3]. Because of the poor prognosis of DIC, it is necessary to identify its potential risk factors. The diagnosis and treatment of DIC are therefore important and an early diagnosis of DIC as pre-DIC may help improve patient survival. Therefore, it is clinically important to identify high-risk patients with DIC promptly and perform the appropriate intervention.

Recently, artificial intelligence has been widely applied to the prediction of various clinical events and including DIC. For example, Yoon et al. [8] exploited several machine learning approaches including logistic regression, linear regression, ridge regression, random forest, and gradient boosting machine to diagnose DIC and prove that machine learning (ML) could optimize the use of clinical parameters for DIC diagnosis. Hasegawa et al. [9] used ML techniques to evaluate predictive accuracies of ML (support vector machine, random forest, and neural network) and conventional approaches (logistic regression) for the progression of coagulopathy in septic patients. It is unearthed in our study that a convolutional neural network (CNN) can provide robust long-term forecasting results in the time-series analysis due to its capability of essential feature learning, distortion invariance, and temporal dependence learning [10].

However, applying CNN to the prediction of DIC based on the densely collected clinical data, especially with the visual interpretation of reasons underlying the prediction results has been scarcely investigated. In this study, we developed CNN to predict the risk of DIC in ICU patients using readily available data from electronic health record (EHR). In addition, Gradient-weighted Class Activation Mapping (Grad-CAM) [11] technique was used to improve the interpretability of the model and to obtain a heat map of the input features.

## Materials and methods

### Data sources

The study cohort included ICU patients from West China Hospital of Sichuan University between January 1, 2019, and January 1, 2022. West China Hospital is a 4300-bed academic hospital in Southwest China and one of the largest hospitals in China. All patient data were obtained from the EHR of the hospital.

### Data selection

**Fig. 1:**
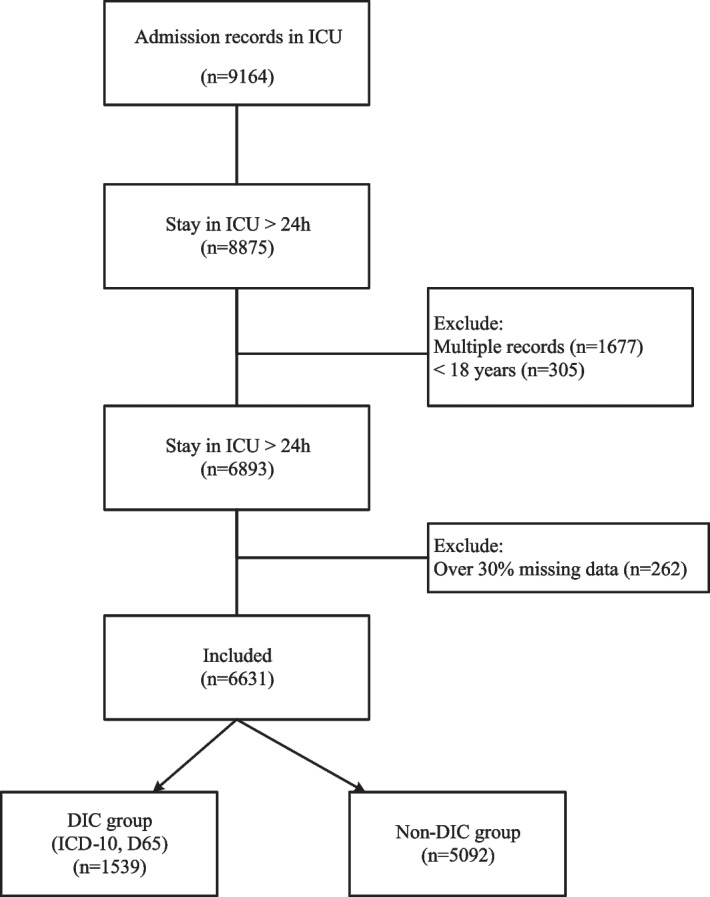
The patients’ selection process

As patients with DIC were the target cohort of this study, we included all the patients who had more than 18 years old and had at least one ICU stay. The diagnosis of DIC (D65) was defined according to the International Classification of Diseases-Tenth Revision (ICD-10) code. Patients with multiple ICU admissions, a hospital stay of ≤ 1 day, or missing data by more than 30% [12] was excluded. The patient selection process was shown in Fig. 1.

### Feature matrix construction

**Fig. 2:**
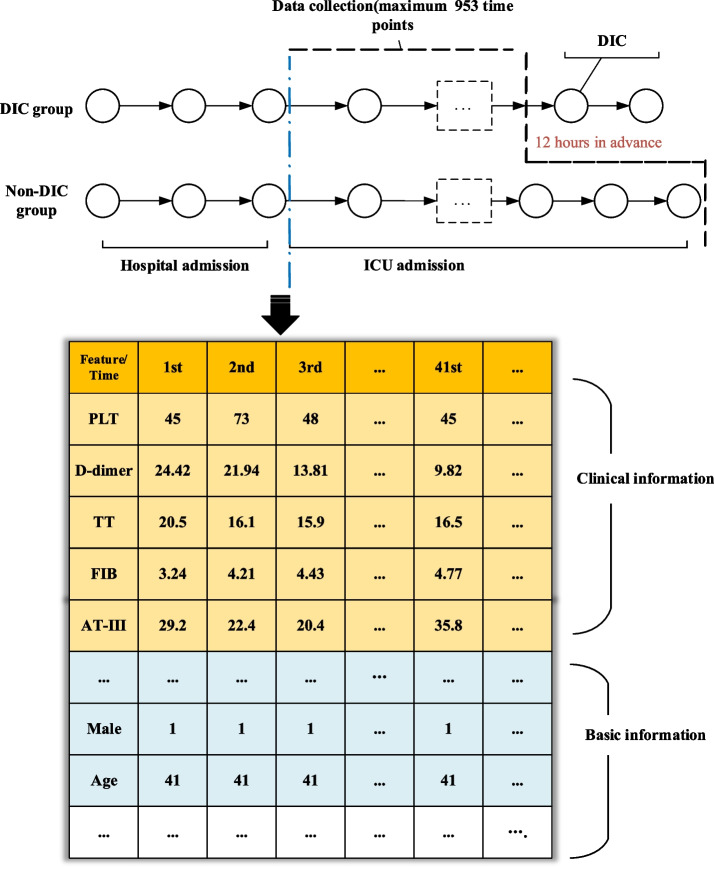
Construction of the feature matrix. (PLT: Platelet, TT: Thrombin time, FIB: fibrinogen, AT-III: Antithrombin III)

We extracted 136 features to predict future ICD based on the EHR, including laboratory test values, demographic characteristics, and clinical events. SQL Server database software and python data preprocessing packages [13–17] were used to sort out the patient time series data matrix. According to the time since the patient entered the ICU, the maximum length of the time series is 953 time steps and the matrix is 953 * 136 [18] (Fig. 2). The data were randomly divided into a training dataset, validation dataset and test dataset in a ratio of 7:2:1.

### Missing data and filling

In time series analysis, missing data is a very common problem in time series [19]. Considering the limitations of traditional missing data imputation methods and the reality of missing data in this study, we adopted the forward imputation method [20] and random forest imputation [21].

### Analysis platform

The current general-purpose deep learning framework PyTorch is used to build predictive models. Every aspect of PyTorch is a normal Python program under the full control of the user [22].

The predictive model was built on a personal desktop computers (operating system: windows 11; central processing unit: 12th Gen Intel(R) Core(TM) i7-12700F; random access memory 32 G; graphics processing unit: NVDIA GeForce RTX3060Ti).

### Artificial intelligence models

#### XGBoost model

XGBoost (Extreme Gradient Boosting) is a scalable machine learning system for tree boosting [21]. It is adept at handling classification and regression tasks. Optimizing the value of the objective function is the core of the algorithm. XGBoost has the advantages of full-scene scalability, speed and performance [23]. Based on the python toolkit (XGBoost Documentation- XGBoost 1.6.1 documentation), we built the XGBoost model. The two-dimensional time series matrix is processed into one-dimensional data into the input model for classification and recognition, where the parameters are set to n_estimators = 50; max_depth = 5; learning_rate = 0.01.

#### LSTM model

**Fig. 3:**
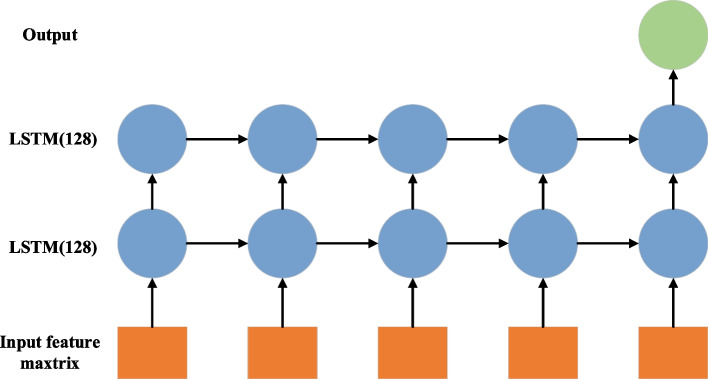
LSTM model

Long short-term memory neural network (LSTM) was developed by Hochreiter and Schmidhuber [24]. It can capture long and short dependencies in time series and is not affected by gradient disappearance [25]. LSTM also has feedback loops, but moreover, it uses a gating mechanism to delete or add information to the model state, and controls model state update and change through the ‘forgetting gate’, ‘input gate’ and ‘output gate’ [26]. It is suitable for processing and predicting events with time series data [25]. We build a Pytorch-based LSTM time series model. The model consists of two layers of LSTMs, each of which consists of 128 hidden units. The binary cross-entropy was used as a loss function and the Adam optimizer was used together with a learning rate of 0.01 (Fig. 3).

#### CNN model

CNN consists of three main neural layers, namely convolutional layers, pooling layers, and fully connected layers that can be stacked according to their functions. CNN make use of knowledge about specific input types rather than focusing on the entire problem domain. This facilitates to set up of simpler network architectures [27]. The advantage of CNN is that it minimizes the number of parameters, which greatly improves the performance of the algorithm [28].

**Fig. 4:**
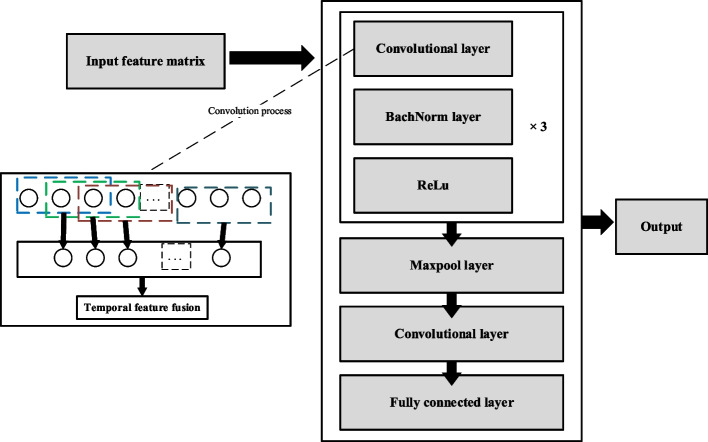
CNN model

In this study, the CNN model adopts the convolution kernel size of 3 * 1 (Fig. 4), only abstracts the features in time series, and retains the feature map consistent with the model input features. This will help to improve the interpretability of models using Grad-CAM technology [10], as well as ensure that the resulting feature maps are consistent with the input features. The maximum pooling layer is used to effectively reduce the size of the parameter matrix and adjust the number of channels and convolution kernel size in the last convolution layer. The 1 * 1 convolution kernel [29] is used for dimensionality reduction and aggregating across channels so that the model gets a feature map with the number of features * 1 dimension before entering the fully connected layer. It can be regarded as a feature of all indicators of the patient. Then the classification result was obtained by inputting the final fully connected layer. The binary cross-entropy was used as a loss function and the Adam optimizer was used together with a learning rate of 0.01. The parameters are shown in Table 1.

**Table 1:** The CNN parameters

Layer	Filters	Size	Stride	Output
Conv1(convolution + BatchNorm + ReLu	16	(3,1)	(2,1)	16 * 476 * 136
Conv2(convolution + BatchNorm + ReLu	32	(3,1)	(2,1)	32 * 237 * 136
Conv3(convolution + BatchNorm + ReLu	64	(3,1)	(2,1)	64 * 118 * 136
Maxpool		(118,1)		64 * 1 * 136
Conv4(convolution + ReLu)	1	(1,1)	(1,1)	1 * 1 * 136
Fully-connected layer	–	–	–	136
Output layer	–	–	–	2

### Model visualization

Gradient-weighted Class Activation Mapping (Grad-CAM) is a visual interpretation technique for decisions from CNN-based models, making them more transparent [10]. It generates location maps using back-propagated gradients of specified class prediction scores to highlight important decision regions of the input matrix. Each feature was fed into the CNN model to generate a predicted score for a class, and the score was back-propagated to the convolutional layer of the model to assign importance values to each input feature. In our study, Grad-CAM uses the gradient information flowing into the last convolutional layer (136 * 1) of the CNN to assign the importance of each feature (neuron) for the decision of interest. Using Grad-CAM, explain how predictive models identify patients with DIC, i.e., determine which features are more likely to trigger patients to develop DIC. The decision-making basis of the model is displayed in the form of a heat map.

The term ${\text{L}}_{{{\text{grad}} - {\text{CAM}}}}^{{\text{C}}} {\mathbb{R}}^{{{\text{u}} \times {\text{v}}}}$ of width u and height v was used to distinguish between DIC and Non-DIC patients. The score gradient was first calculated for class c and ${\text{y}}^{{\text{c}}}$ (before SoftMax), with respect to feature maps ${\text{A}}^{{\text{k}}}$ in the final convolutional layer. These gradients were global average pooled to obtain neuron importance weights ${\text{a}}_{{\text{k}}}^{{\text{c}}}$:

1 $${\text{a}}_{{\text{k}}}^{{\text{c}}} = \frac{1}{{\text{z}}}\mathop \sum \limits_{{\text{i}}} \mathop \sum \limits_{{\text{j}}} \frac{{\partial {\text{y}}^{{\text{c}}} }}{{\partial {\text{A}}_{{{\text{ij}}}} }},$$

representing the importance of a feature map k in a target class c. A weighted combination of forwarding activation maps was then performed, followed by a ReLU, to obtain the final heat map:

2 $${\text{L}}_{{{\text{grad}} - {\text{CAM}}}}^{{\text{C}}} = {\text{ReLU}}\left( {\mathop \sum \limits_{{\text{k}}} {\upalpha }_{{\text{k}}}^{{\text{c}}} {\text{A}}^{{\text{k}}} } \right).$$

In a deep network, heat maps can be used to visualize any area that contributes to classification results, thereby increasing interpretability, as was the case in this study.

### Statistics

Categorical variables are presented as counts and percentages, and continuous variables are presented as mean and standard deviation (SD). Comparisons between groups were performed by 2-tailed t-test for continuous variables and chi-square test for categorical variables. All statistical analyses were performed in the python package SciPy (SciPy) [30]. The statistical significance was considered as *P* < 0.05.

## Results

### Patient characteristics

The cohort included 6631 ICU patients, of whom 1539 patients (23.2%) developed DIC. There were more males than females in this cohort (DIC: 60.0% male vs 40.0% female; Non-DIC: 60.2% male vs 39.8% female). The mean age of DIC patients and Non-DIC patients was 52.6 and 51.3 years old, and BMI was 21.88 and 22.79, respectively. There was no statistical significance (*P* > 0.05). The basic demographic characteristics of the cohort was shown in Table 2.

**Table 2:** Base characteristics of the included patients

Variables (mean ± SD)	DIC	Non-DIC	*P*
Sex			> 0.05*
Male	923 (60.0%)	3069 (60.2%)	
Female	616 (40.0%)	2023 (39.8%)	
Age (years)	52.60 ± 21.24	51.35 ± 23.25	> 0.05
BMI	21.88 ± 4.49	22.79 ± 4.35	> 0.05
Surgery	842	4443	< 0.001
Ventilator use	684	718	< 0.001
ICU stay	10.76 (13.20)	5.77 (11.33)	< 0.001

*Chi-square test

### Model performance

**Fig. 5:**
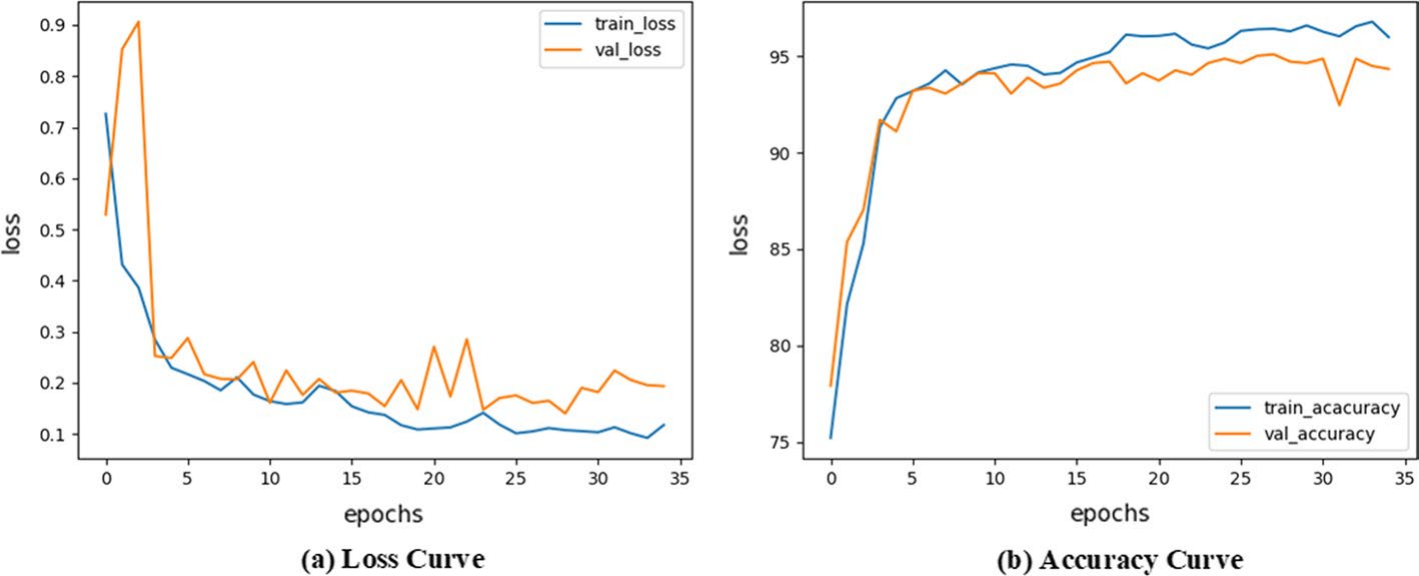
Loss and accuracy curve for the CNN model

For the CNN model, the Loss value decreases gradually with epoch and stabilizes at 35 epochs, and the accuracy reaches 95% on both the training and validation sets (Fig. 5).

**Fig. 6:**
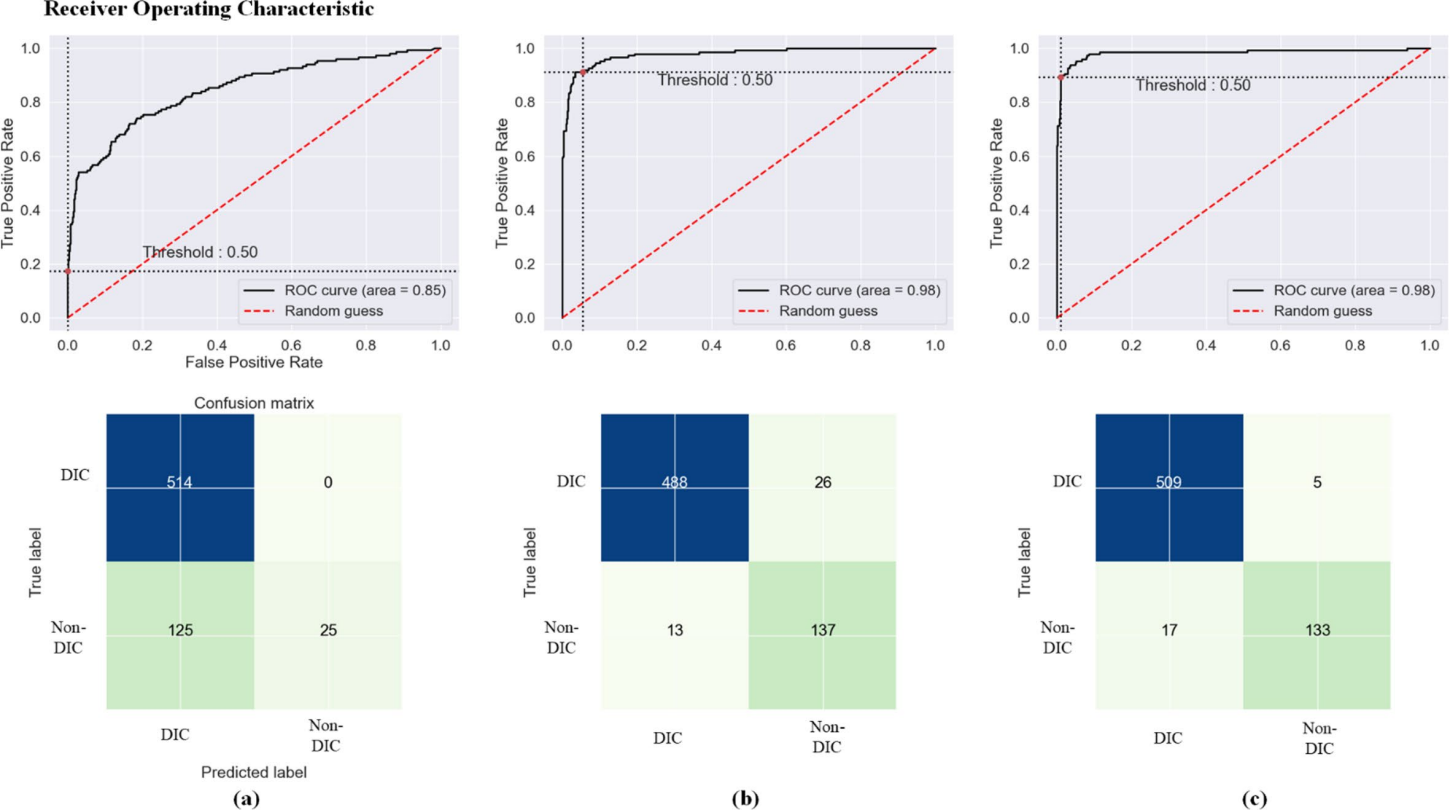
ROC and confusion matrix of three prediction models (**a** XGBoost; **b** LSTM; **c** CNN)

Figure 6 shows the area under the receiver operating characteristic (AUROC) curves for these predictive models. Among the three models, the CNN model showed the best area under the curve (AUC) (0.986), accuracy (95.7%) and F1 (0.935), which was statistically significant with XGBoost (*P* < 0.01) (Table 3).

**Table 3:** Comparison of prediction performance among the three models

Model	XGBoost	LSTM	CNN	*P* (XGboost vs. LSTM)	*P* (XGboost vs. CNN)	*P* (CNN vs. LSTM)
AUC	0.851 (0.035)	0.983 (0.022)	0.986 (0.018)	< 0.001	< 0.001	> 0.05
Accuracy	82.03% (1.63%)	95.13% (0.69%)	95.68% (0.71%)	< 0.001	< 0.001	> 0.05
F1-score	0.771 (0.058)	0.915 (0.061)	0.935 (0.044)	< 0.001	< 0.001	0.0197

### Model interpretation

**Fig. 7:**
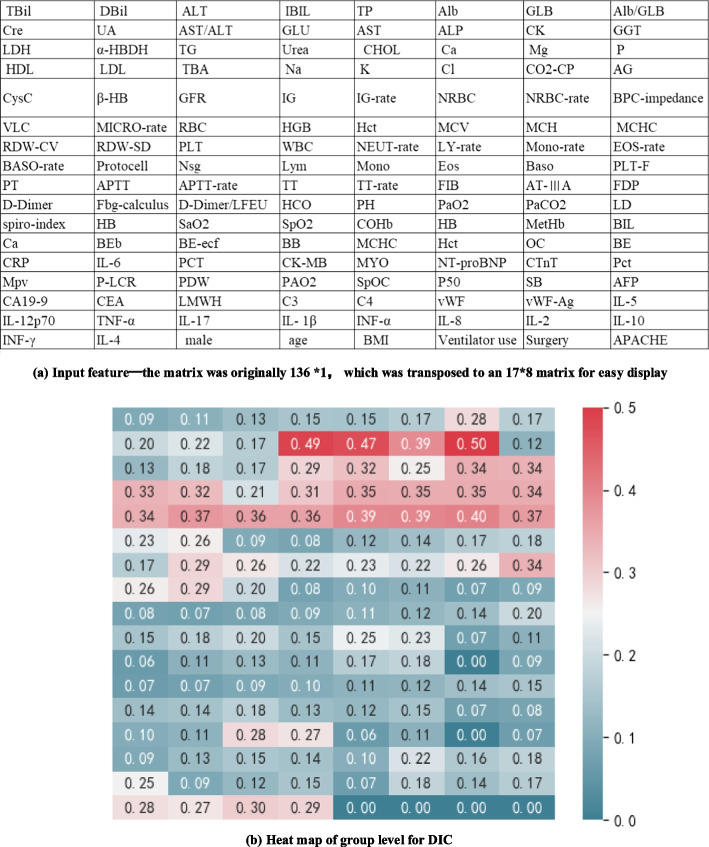
Input feature and heat map

To understand the contribution of features to the model predictions, Grad-CAM was used to interpret the CNN predictions, as shown in Fig. 7. Figure 7a showed the 136 features of the input model (Additional file 1); Figure 7b showed the activation features on all samples in the CNN model. Grad-CAM analysis identified that the top ten features for DIC prediction were CK (creatine kinase), GLU (glucose), AST (aspartate aminotransferase), NRBC-rate (nucleated red blood cells rate), NRBC, IG-rate (immature granulocyte rate), ALP (Alpha-fetoprotein), β-HB (β hydroxybutyric acid), BPC-impedance (blood platelet count), IG (immature granulocyte). The higher the value of Grad-CAM, the higher the risk of model output (increased risk of DIC).

**Fig. 8:**
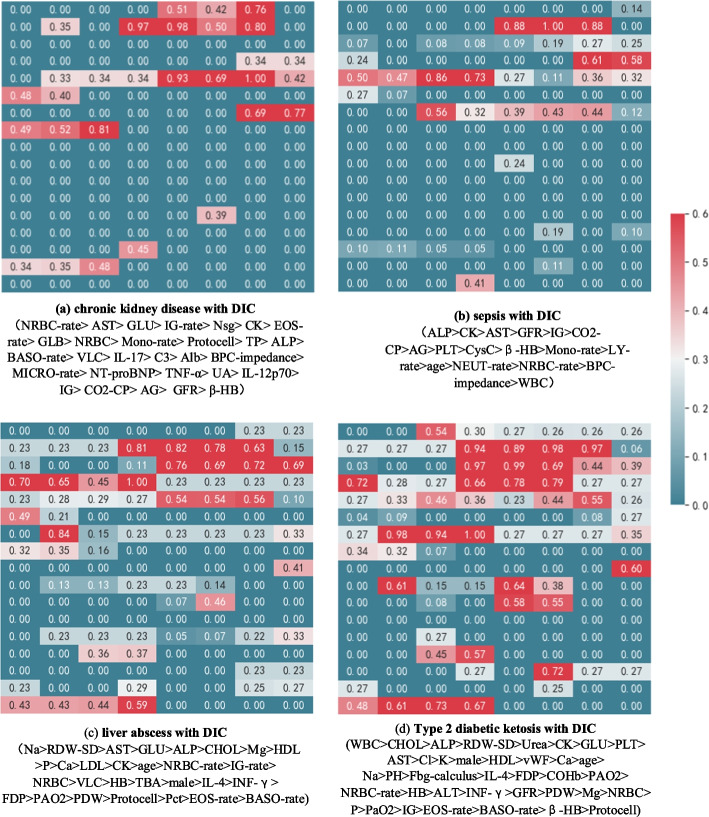
Heat map of different diseases

Figure 8 showed an example of real-time sequential prediction using the CNN model on different patients (chronic kidney disease, liver abscess, sepsis, type 2 diabetes ketosis). With data from each time point after a patient enters the ICU, the model provides a real-time assessment of the risk and uncertainty of future DIC episodes. This demonstrates that the model can detect DIC up to 12 h in advance, which is important for clinicians to take preventive action before an event occurs.

## Discussion and conclusions

DIC represents the end-stage of different coagulation disorders. It is a complex syndrome involving the dysfunction of multiple physiological systems and processes. The characteristics of the multiple coagulopathies described may differ considerably depending on the pathophysiology and time in the disease course.

In our study, DL and ML algorithms were used to perform early prediction of DIC. The patient’s temporal data during ICU is processed into a two-dimensional matrix, and a convolutional network is used to convolve the temporal sequences on each dimensional feature. This can also be seen as combining features from different temporal points or convolving the receptive field of the kernel to perform feature abstraction over the temporal sequence. In addition, the data size and model complexity in our study are not high, and both RNN and CNN networks can obtain good classification performance for such a size study. The results show that the development of models to identify patients who are at high risk of developing DIC early in ICU patients is clinically important to predict the prognosis. The results might be explained by the time window for the anticoagulant therapy [31], since the use of anticoagulation at an early stage may be most effective [32, 33]. Early diagnosis of DIC is important for the restoration of coagulation abnormalities and patient survival [34]. Thus, the prediction of coagulopathy development is clinically important for the proper selection of patients and anticoagulation time window.

In order to facilitate clinical application and help clinicians to understand, CNN models are visualized. Combined with Grad-CAM technology, the important features that affect decision-making are displayed in the heat maps [35]. The CNN model not only explains the decision-making process of deep learning models but also provides visualization of the feature selection process.

Since DIC is an acquired syndrome, various underlying causes may influence the clinical manifestations of DIC and may produce different accents on the laboratory findings [36, 37]. A group-level activation heat map was obtained by Grad-CAM, and some differences in activators of DIC induced by different diseases could be found (Fig. 8). Although the findings demonstrate good performance and accuracy of the CNN prediction model, it still needs multi-centre validation before it can be used in clinical practice.

However, our study also has some limitations. First, this model was developed at a single center, which reduces effectiveness and may require retraining when applying the model to other hospitals. Secondly, the results of different DIC risk predictions are often difficult to compare due to their different data sources, inconsistent data inclusion criteria, and differences in data types. In addition, the study was retrospective and requires further validation in prospective clinical studies. In future studies, more variables such as medications [38] and protein sequences [39] will be used in early prediction models for DIC.

In the study, we propose a CNN Grad-CAM model for the early prediction of DIC. The model uses Grad-CAM technology to visualize and interpret the prediction results. It provides a faithful visual explanation of the decisions of the DIC prediction model. Further studies should conduct external validation of the model to ensure its suitability for clinical application.

## Supplementary Information

**Additional file 1.** Related acronyms and abbreviations.

## Data Availability

The data that support the findings of this study are available from the corresponding author upon reasonable request (Dljl8@163.com).
