# Supplementary material for: An interpretable DIC risk prediction model based on convolutional neural networks with time series data
Source: BMC Bioinformatics. 2022 Nov 8;23:471. doi: 10.1186/s12859-022-05004-2 (PMC9644626; doi:10.1186/s12859-022-05004-2)
Supplement: Supplementary file 1 — Additional file 1. Related acronyms and abbreviations. [file 12859_2022_5004_MOESM1_ESM.docx]

Additional file 1

Supplementary

TBil:Total bilirubin; DBil:Direct bilirubin; ALT:Alanine aminotransferase; IBIL:Indirect

bilirubin; TP:Total protein; Alb:albumin; GLB:globulin; Cre:creatinine; UA:uric acid; GLU:glucose; AST:Aspartate aminotransferase; ALP:alkaline phosphatase; CK:creatine kinase; GGT:Glutamyl transpeptidase; LDH:lactate dehydrogenase; α-HBDH:Hydroxybutyrate dehydrogenase; TG:Triglycerides; Urea:ureophil ; CHOL:cholesterol; HDL:high-density lipoprotein; LDL:Low-density lipoprotein; TBA :Total bile acid ; CO2-CP:Carbon dioxide binding; AG:Anion gap; CysC:Serum cystatin C measurement; β-HB: β hydroxybutyric acid ; GFR: glomerular filtration rate; IG: immature granulocyte; IG-rate:immature granulocyte rate; NRBC:Nucleated Red Blood Cells; NRBC-rate: Nucleated Red Blood Cells rate; BPC-impedance:Impedance method platelets; VLC:Mutant lymphocyte; MICRO-rate: Percentage of small red blood cells; RBC: Red blood cell count; HGB:haemoglobin; Hct:Hematocrit; MCV:mean corpuscular volume ; MCH :meam hemoglobin content of erythrocytes; MCHC :meam red blood cell hemoglobin concentration; PLT:Platelet; WBC:white blood cell; NEUT-rate:Neutrophil rate; LY-rate:lymphocytes rate; Mono-rate: Monocyte rate; EOS-rate:Eosinophil rate; BASO-rate:Basophil rate ; Protocell:Protocell; Nsg:neutrophilic segmented granulocyte; Lym:Lymphocyte; Mono: Monocyte ; Eos:Eosinophil; Baso:Basophil; PLT-F:Low channel platelets; PT:Prothrombin time; APTT:Activates partial thromboplastin time; APTT-rate:Activated partial thromboplastin time tate; TT:Thrombin time; TT-rate:Thrombin time rate; FIB:fibrinogen; AT-ⅢA:Antithrombin III; FDP:Fibrin and fibrinogen degradation products; D-Dimer:D-Dimer; Fbg-calculus:Fibrinogen calculus; LD: lactic acid; HB:hemoglobin; COHb:carboxyhemoglobin; HB:hemoglobin; MetHb:Methemoglobin; BIL:bilirubin; BEb:base excess blood; BE-ecf:base excess extracellular fluid; BB:Buffer base; MCHC:Mean corpuscular hemoglobin concentration; Hct:Hematocrit; OC:oxygen capacity; BE:base excess; CRP:C-reactive protein; IL-6:Interleukin-6; PCT:Procalcitonin; CK-MB:Creatine kinase isoenzyme MB; MYO:myoglobin; NT-proBNP:Type B natriuretic peptide precursor; CTnT:Troponin-T; Pct:Platelet hematocrit; Mpv:mean platelet volume ; P-LCR: percent of Large platelet; PDW:Platelet distribution width; PAO2:Partial Pressure of Oxygen; P50:Hemoglobin hemisaturated oxygen partial pressure; SB:Standard bicarbonate concentration; AFP:Alpha-fetoprotein; CA19-9:carbohydrate antigen 19-9; CEA:Carcinoembryonic antigen; LMWH:Anti-Xa (low molecular weight heparin); C3:Complement C3; C4:Complement C4; vWF:von Willebrand factor activity; vWF-Ag:von Willebrand factor antigen; IL-5:Interleukin-5; IL-12p70:Interleukin; TNF-α:Tumor necrosis factor α; IL-17:Interleukin; IL- 1β:Interleukin; INF-α:interferon α; IL-8:Interleukin-8; IL-2:Interleukin-2; IL-10:Interleukin-10; INF-γ:interferon γ; IL-4:Interleukin-4; BMI :Body Mass Index;）
